# An Automatic Instrument Integration Scheme for Interoperable Ocean Observatories

**DOI:** 10.3390/s20071990

**Published:** 2020-04-02

**Authors:** Shijun Lin, Feng Lyu, Huixin Nie

**Affiliations:** 1 State Key Laboratory of Marine Geology, Tongji University, Shanghai 200092, China; linshijun@tongji.edu.cn (S.L.); niehx@tongji.edu.cn (H.N.); 2 School of Ocean and Earth Science, Tongji University, Shanghai 200092, China; 3 Center for Marine Science and Technology, Tongji University, Shanghai 200092, China

**Keywords:** ocean observatories, science instruments, automatic integration, machine-to-machine interoperability

## Abstract

Due to the heterogeneity, high cost, and harsh environment, ocean observatories demand a flexible, robust, and capable scheme to integrate science instruments. To deal with the challenges of automatic instrument integration and machine-to-machine interaction in ocean observatories, a systematic scheme is proposed based on Zero Configuration Networking (Zeroconf), Programmable Underwater Connector with Knowledge (PUCK), Constrained Application Protocol (CoAP), and Message Queuing Telemetry Transport (MQTT) protocols, as well as a smart interface module to achieve instrument plug-and-play and standard communication among heterogeneous ocean instruments. The scheme specifically considers the resource-constrained ocean observatories and machine-to-machine interoperability, which is of great significance to interoperable ocean observatories. The laboratory tests have verified the feasibility of the proposed scheme.

## 1. Introduction

**Figure 1:**
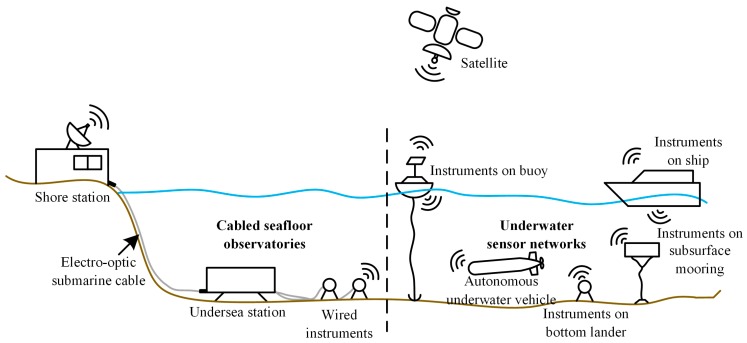
Two typical systems of scientific ocean observatories.

Scientific ocean observatories, including cabled seafloor observatories and underwater sensor networks as shown in Figure 1, have provided various oceanographic instruments with power supply and communication bandwidth for science data collection in recent years. Cabled seafloor observatories supply ocean instruments with abundant power and bandwidth from shore stations through electro-optic submarine cables [1,2]. The constraint in this scenario is the computing capability of embedded microcontrollers in undersea equipment. Acoustic underwater sensor networks consist of instruments fixed on the seafloor, in the water body, on the surface, and in the moving vessels communicating with one another wirelessly [3,4]. They have additional limits in terms of power supply and the connectivity to the shore station. Due to the low bandwidth and high delay of acoustic communication, the connectivity among the instruments is also limited. Moreover, the heterogeneity, high cost, and harsh environment make it a great challenge to integrate a large number of instruments.

**Figure 2:**
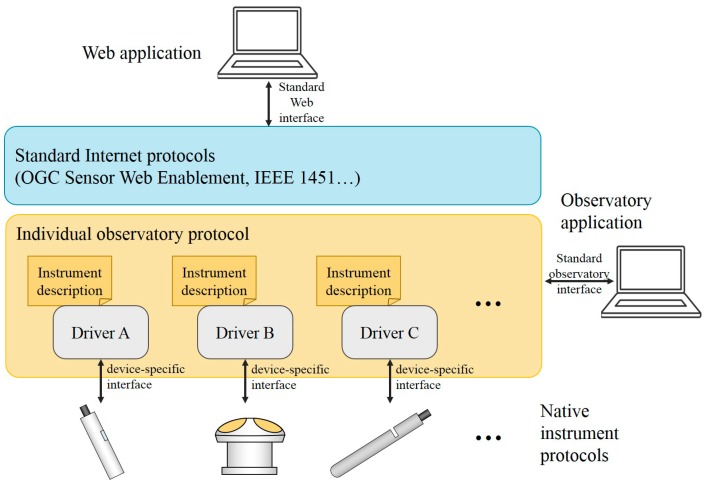
Interoperable instrument protocol layers [5].

In traditional design, each instrument interface of an ocean observatory is customized depending on the connected instrument. This solution is acceptable for small-scale observatories with just a few fixed instruments. However, large-scale observatories need a flexible scheme to integrate new instruments and to facilitate interoperation within the observatory system, i.e., to communicate and exchange data among instruments. The key to achieving this is interface standardization, i.e., to unify the hardware and software interfaces of ocean instruments in the system. Since there is no common standard for ocean instruments, the current solution is adding a protocol layer to the system architecture of an ocean observatory [5], as shown in Figure 2. Drivers are customized for each instrument type to translate between native instrument protocols and individual observatory protocol with description files [6]. In this way, every nonstandard instrument is standardized on the individual observatory protocol layer by mapping between the two protocols. IEEE 1451 and Open Geospatial Consortium—Sensor Web Enablement (OGC-SWE) have been adopted to facilitate ocean instrument interoperation [7,8,9]. IEEE 1451 defines a set of open, network-independent communication interfaces [10], but it is complicated and lacks flexibility. OGC-SWE defines an open standard framework to exploit sensors or instruments via the internet for worldwide data exchange [11], but it is also very complicated and unsuitable for resource-constrained underwater scenarios.

Several integration schemes have also been proposed. Gigan et al. proposed the Sensor Abstract Layer (SAL) to aggregate multiple sensor networks and provide a generic, hardware-independent interface to manage sensors [12]. Yu et al. implemented a plug-and-play monitoring system, in which a centralized and configurable host software is deployed onshore to manage all instruments [13]. The NEPTUNE project developed an Enterprise Service Bus (EBS)-based data management and archiving system (DMAS) to real-time interact with instruments and manage data [14]. However, every instrument requires manual configuration to bind itself with its description file and its driver once it is reintegrated into the system. 

In order to achieve automatic instrument integration, an additional firmware middleware named smart interface modules has been adopted by some ocean observatories in recent years. Monterey Bay Aquarium Research Institute (MBARI) developed a Programmable Underwater Connector with Knowledge (PUCK) firmware to carry instrument metadata [15,16,17] for integrating instruments into their JAVA-based cyberinfrastructure. However, Java requires considerable computing and communication resources [18]. The PUCK firmware has also been evaluated to combine with IEEE 1451 and OGC-SWE standards [5,6,19,20]. The NeXOS project developed the Smart Electronic Interface for Sensors and Instruments (SEISI) to enable web-based sharing, discovery, exchange, processing, and operation of ocean observations [21]. Considering the high resource requirements of the web service, we proposed the Smart Instrument Adapter (SIA) for localized integration in an ocean observatory [22], that has great value when the connectivity between the instruments and the center node is limited. Moreover, machine-to-machine communication specially considered by SIA can also facilitate cooperation among instruments. A comparison of these schemes is given in Table 1. However, automatic instrument integration still lacks enough studies for interoperable ocean observatories. 

This paper is organized as follows. In Section 2, the challenges and requirements of automatic instrument integration for interoperable ocean observatories are summarized and our scheme is introduced, based on our SIA and selected existing standards. In Section 3, a prototype of the proposed scheme and its laboratory test are presented to verify the feasibility of the design. Finally, Section 4 concludes the paper.

## 2. Design of the Automatic Instrument Integration Scheme

The proposed scheme is designed above the network layer, which means the wired or wireless TCP/IP networking is assumed established, and the scheme covers the transport layer and session layer, referring to ISO/OSI seven-layer model. 

### 2.1. Challenges and Requirements

The goal of the scheme is not only to enable the automatic integration of various instruments for the control and data center, but also to support machine-to-machine interaction so that instruments can request services from one another, and cooperate without the intervention of the center node, which suggests great value where the connectivity to the center node is low. The challenges can be summarized as below.

(1) The architecture of the scheme should standardize various instruments with different intelligence levels and different native protocols.
(2) The scheme should enable reliable networking in the absence of external configuration information, which means each instrument can be automatically allocated a valid network address which can be resolved by the domain name, even if there are no DHCP or DNS servers in the local network.
(3) Instruments in the same network should be able to detect the services of other ones with no external assistance.
(4) Each instrument should be uniquely identified throughout its whole lifetime so that it can be recognized after it is physically removed/added, or its network address is changed.
(5) Entities in an ocean observatory should interact with others by standardized and uniform processes, so that software and hardware modules can be reused to save cost.
(6) Considering the limited resources and capability of embedded devices used in ocean observatories, as well as the limited power availability, low-bandwidth communications, and high maintenance costs, the protocols adopted should be energy and bandwidth saving.
(7) For better interoperability among instruments, existing standard protocols should be used whenever possible.

To deal with the above challenges, the design of the scheme should meet the following requirements.

(1) Standard Interface: Nonstandard instruments should be converted to the standard interface.
(2) Automatic Configuration: Instruments should complete network configuration independently so that they can communicate with one another in a temporary local network.
(3) Service Detection: After instruments have been ready for communication, they should be able to announce their service and to find out where to get the services of others.
(4) Service Identification: The description of the instrument services should be obtained to know what the services are and to identify the same one when the network address changed.
(5) Standard Session: Instruments should follow standard procedures to establish sessions with peers to exchange data or send/receive commands. Request/response mode and subscribe/publish mode should be supported.

### 2.2. Protocols and Standards

**Figure 3:**
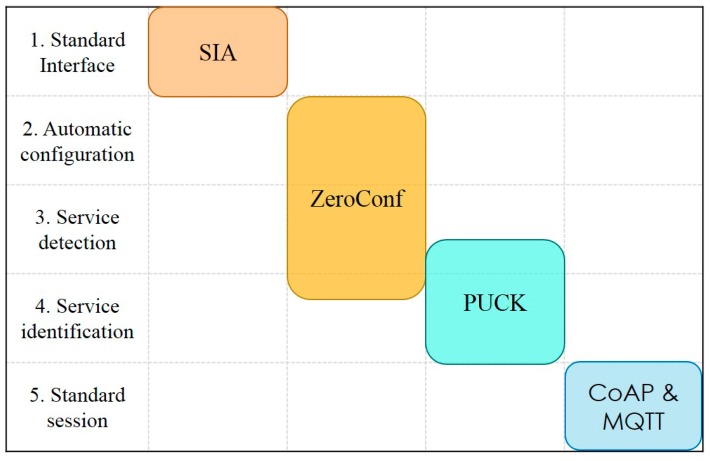
Protocols and standards adopted in the proposed scheme.

The protocols and standards used in our scheme are shown in Figure 3; the SIA firmware, as well as the Zero Configuration Networking (Zeroconf), PUCK, CoAP, and MQTT protocols, are adopted to meet the designed requirements.

#### 2.2.1. SIA

**Figure 4:**
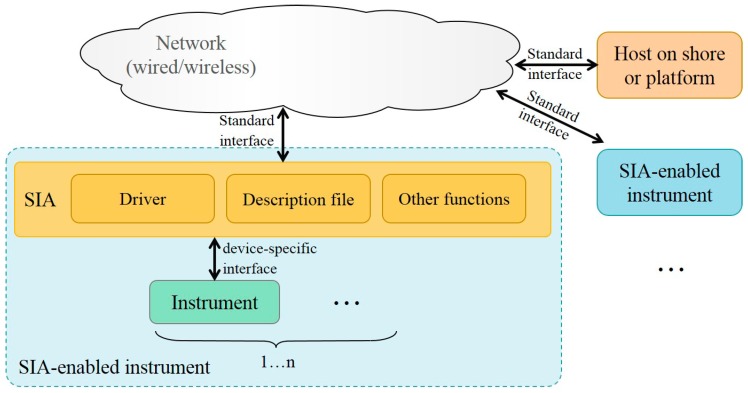
The diagram of SIA-enable instruments [22].

The SIA is used to adapt instruments with nonstandard interfaces to standard interfaces. The concept of SIA is shown in Figure 4. The SIA works as a communication middleware to translate the native instrument protocol to standard observatory protocols, so that standardization is achieved at the instrument-side. Although the driver needs to be customized for each instrument type, the software interfaces for network services and basic functions can be reused. SIA-enabled instruments are featured by distributed intelligence and “peer-to-peer” characteristics, which benefit machine-to-machine interaction.

#### 2.2.2. Zeroconf

The Zeroconf protocols are adopted to enable automatic configuration, service detection and part of service identification. Zeroconf defines three sub-protocols [23]. The IPv4 Link-Local (IPv4LL) protocol defines how to configure the network automatically when there is no external information, e.g., DHCP server, so that instruments can communicate among one another in a link-local network. The Multicast DNS (mDNS) protocol specifies how to perform DNS queries over IP multicast and how to make up a local domain name so that instruments can be assessed by a human-readable domain name, but not an IP address, without the help of a DNS server. In our scheme, each SIA is assigned a unique local domain name. The DNS Service Discovery (DNS-SD) protocol, based on standard DNS programming interfaces, servers, and packet formats, provides a way to browse the network for services. It is compatible with mDNS, and it is used over mDNS to achieve service detection in the scheme. 

#### 2.2.3. PUCK

The PUCK protocol is implemented in our SIAs so that instruments can be detected and identified. This protocol defines the format of an internal memory, which consists of predefined minimal metadata and an optional “payload”, and a simple communication protocol to access it [24]. The predefined metadata includes UUID for instrument, version of instrument datasheet, datasheet size, manufacture ID, manufacture model, manufacture version, serial number and instrument name, which is enough to identify an instrument. The content in the payload is completely arbitrary so that any metadata standards can be used as needed to store extended metadata for software interfaces and so on. The information to be stored in the PUCK memory is referred to as the Transducer Electronic Data Sheet (TEDS) in the following sections.

Clients use DNS-SD to find the PUCK service, then retrieve the metadata to know which one and what type of the instrument is, and to figure out how to communicate with the instrument without any prior knowledge of it. 

#### 2.2.4. CoAP

The CoAP protocol is a specialized web transfer protocol for resource-constrained nodes and networks [25], which is based on the widely successful Representational State Transfer (REST) model. Every resource is available under a Uniform Resource Locator (URL), and clients access these resources using commands such as GET, PUT, POST and DELETE. The scheme adopts it for request/response sessions. 

#### 2.2.5. MQTT

The MQTT protocol is an extremely lightweight publish/subscribe messaging transport [26], adopted by the proposed scheme for publish/subscribe sessions when a broker can be deployed. It is ideal for embedded devices because of its low power consumption, minimized data packets size, and efficient data distribution. In the MQTT, a TCP server called broker plays the role of the message center to distribute messages from and to MQTT clients, and clients connect to the broker to publish or subscribe message. MQTT message channels are identified by the topic name. A client publishes a message of a topic, and then the message will be transmitted to all clients who have subscribed to the topic in real-time.

### 2.3. Scheme Design

The traditional design of ocean observatories uses centralized architecture, a center node works as a master, and all instruments work as slaves to serve it. However, the proposed scheme is designed based on the Service-Oriented Architecture (SOA) concept, in which every object is considered as a service entity. Each service can advertise itself on the network so that peers can find it and request service. The comparison of two architectures for ocean observatories is presented in Table 2. The high scalability of SOA facilitates the integration and machine-to-machine interoperability. In an ocean observatory based on this scheme, a master node will be deployed for management.

**Figure 5:**
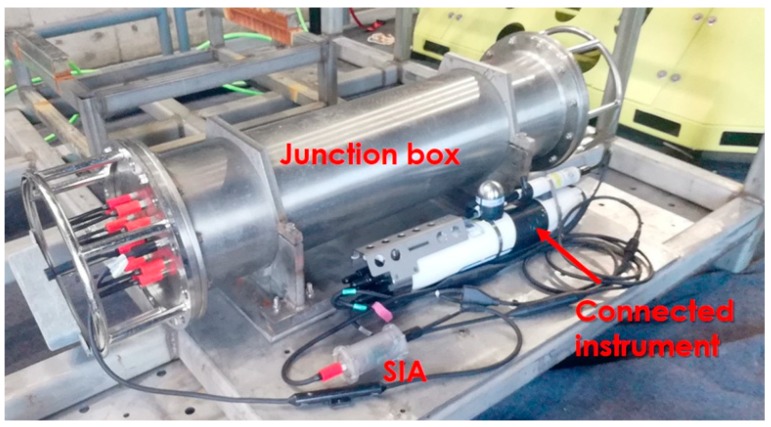
Using the SIA as middleware between a junction box and an instrument.

In our scheme, each nonstandard instrument is connected with an SIA external module, as Figure 5 shows. The SIA module can also be installed inside the instrument. The underwater junction box links to the undersea station and provides standard interfaces with DC 48V power output and Ethernet communications so SIA hardware and software of network-side can be mostly standardized, but instruments have various interfaces with different low voltage inputs and different communication interfaces so the instrument-side is responsible for stepping down the voltage from the junction box and converting communication interfaces to Ethernet communications for the connected instrument.

On the instrument-side, each SIA hardware needs a DC–DC converter to provide suitable power supply voltage. To adapt various communication interfaces, the SIA hardware reserves some common electronic interfaces so that the appropriate interface can be chosen as needed.

Ocean instruments have digital or analog interfaces. Because of the diverse instrument interfaces, it is impossible to develop a universal driver for all instrument types, so the solution is to leave the interface adaptation issue to the customized drivers. For an analog instrument, the driver uses an analog to digital converter to sample the analog signal and calculate scientific seawater parameters. Digital instruments typically apply EIA-232, EIA-485, CAN or Ethernet interfaces, using customized native instrument protocols to get commands and output the data. For a digital instrument, the driver is developed to communicate with it on the corresponding electronic interface with its native instrument protocol. Moreover, I/O pins of MCU can be connected with it, so that the driver can control the instrument directly.

On the network-side, the hardware is standardized to use the Ethernet interface and fixed input voltage and the software needs to have the MQTT client, CoAP server, Zeroconf proxy, PUCK server module and other universal modules, which can be reused in all SIAs. Based on the universal modules, the driver code should be customized for each instrument type to provide services over the network by the standard scheme. 

Once the driver has been ready for an instrument type, the main task for integrating a new instrument of the same type is to prepare a new SIA firmware, connect the corresponding hardware interfaces, fill in a unique TEDS and modify some configuration settings for it so that peers can identify it and its software can produce derivative configuration automatically, e.g., service instance name and host domain name. 

Once powered up, the SIA first initializes all universal modules and registers TEDS to the PUCK module. The PUCK server starts listening on the TCP port and registers the service instance to the Zeroconf proxy module. Then, the Zeroconf proxy advertises the service instance and listens over mDNS. In the meantime, the CoAP server starts listening on the UDP port, and the port number is registered to the PUCK module. If an MQTT broker is available, the MQTT client module will figure out the address of the MQTT broker and connect with it, waiting for publishing data for the driver. 

After the universal modules ready, the SIA runs the driver code and communicates with the instrument for initialization and regular data collection tasks if necessary. Then, the driver registers every resource and corresponding call-back interface to the CoAP server. Whenever a request comes from the CoAP module, the driver executes the job and returns the result. Once data collection is completed, the driver analyzes and packets the data in a predefined format, and sends the data to the MQTT or CoAP module for publishing. In addition, the driver can provide the instrument with other advanced functions, e.g., data storage, timing task, and data calibration. 

**Figure 6:**
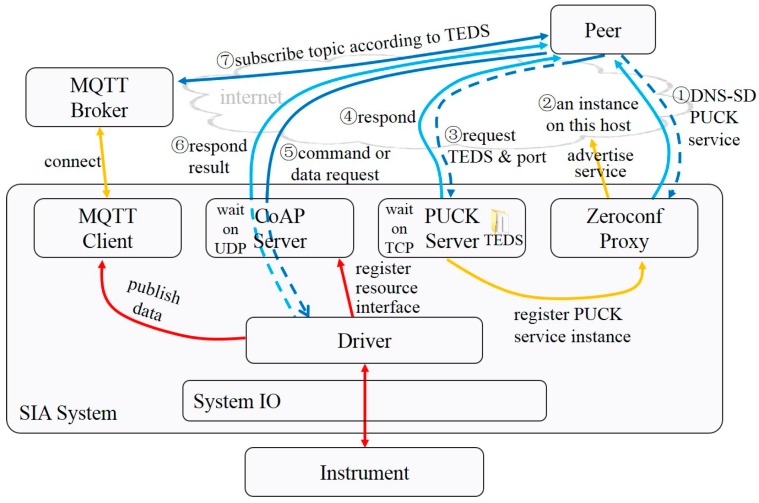
Interoperable communication scheme with MQTT broker.

The scheme for the common scenario, where an MQTT broker is available, is shown in Figure 6.

The common interaction process for a peer to get services from a networked service instance with no prior knowledge in this scenario is summarized below.

① The DNS-SD protocol is applied to browse the PUCK service over mDNS.

② The Zeroconf proxy of the SIA responds with the domain name and port of the PUCK Server, once it receives the request message. Then, the peer gets a list of available service instances.

③ The peer chooses the wanted instance. TCP connects to the PUCK server, reads the TEDS, and asks for the port of the CoAP server.

④ The PUCK Server of the SIA receives the request, then responds.

⑤ The peer figures out the URLs of resources, according to the predefined patterns and information in the TEDS. When a peer wants to control the instrument or request one-shot data, it sends a request to the CoAP server to access the resource.

⑥ The CoAP Server of the SIA translates the request, interacts with the instrument to execute the command, and responds results.

⑦ The peer figures out the topic names the service instance will publish data to, according to the predefined pattern and information in the TEDS, and subscribes real-time data over the MQTT broker pre-connected.

**Figure 7:**
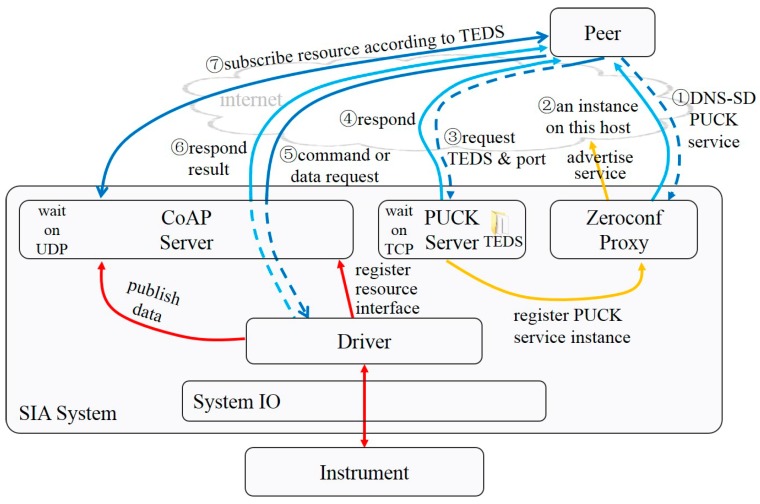
Interoperable communication scheme with no MQTT broker.

The scheme for the common scenario, with no MQTT broker available, is shown in Figure 7.

The common interaction process for a peer to get service from a networked service instance with no prior knowledge in this scenario is almost the same, except for ⑦, presented below.

⑦ The peer figures out the URL of resources, according to predefined pattern and information in the TEDS, and subscribes real-time data from the CoAP server.

In this way, the scheme enables the plug-and-play integration of ocean instruments and instruments can detect and cooperate with each other.

## 3. Implementation of the Scheme for Interoperable Ocean Observatories

### 3.1. Hardware Implementation

**Figure 8:**
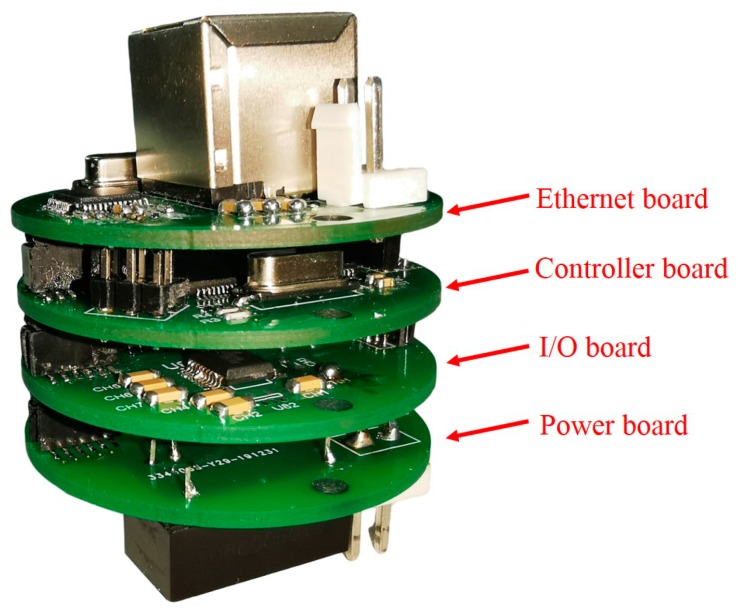
Multi-layer stacked printed circuit boards of the SIA.

As Figure 8 shows, the printed circuit board of the SIA is designed as a multi-layer stacked structure, including an Ethernet board, a controller board, an I/O board, and a power board. The Ethernet board contains network chips and electronic interfaces. The chip WIZnet W5500 is used to provide a hardware TCP/IP protocol stack. The controller board contains a microcontroller, a storage chip, and a clock chip. The microcontroller is NXP MC9S12 which provides abundant standard on-chip peripherals [27]. The I/O board contains chips and interfaces for interacting with instruments, supporting serial communication interface EIA-232 and EIA-485, as well as an analog to digital conversion function. The power board steps down the input voltage to power the SIA and the instrument with a suitable DC–DC converter.

The multi-layer stacked structure design provides an easy way to replace or obtain functions. For example, the power board can be replaced to provide different power supply voltages for the connected instrument and the I/O board can be replaced to get other electronic interfaces as needed.

### 3.2. Software Implementation

**Figure 9:**
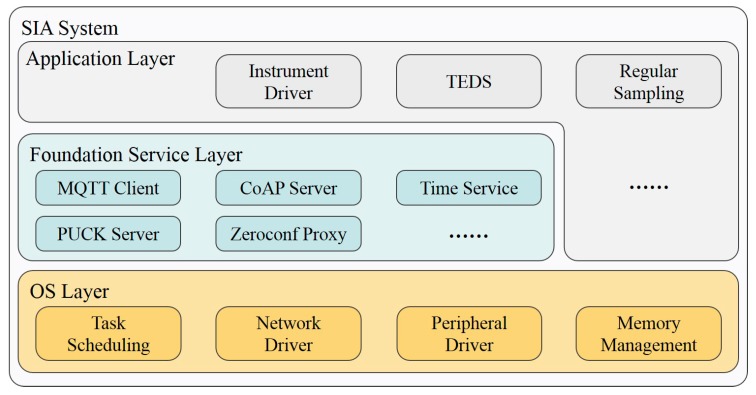
The software architecture of the SIA.

The SIA software is developed by C language in CodeWarrior, which is the official IDE of MC9S12, and based on the uCOS-II real-time operating system, to maximize utilization and real-time performance. The SIA software has the OS layer, foundation service layer, and application layer. Each function is encapsulated into a module, as shown in Figure 9.

The OS layer provides essential functions, e.g., task scheduling, memory management, and peripheral driver. Uniform software interfaces of peripherals and Berkeley Software Distribution (BSD) style network interfaces are provided to extend uCOS-II kernel to get a feature-rich OS layer, serving the upper layers. 

The foundation service layer is mainly for SIA functions based on the OS layer. The modules for the standard communication scheme, i.e., MQTT Client, CoAP Server, PUCK Server, and Zeroconf Proxy, are all in this layer. They provide upper application configuration interfaces to register device-specify information and resources, and communication interfaces to publish real-time data. Most of them create tasks to serve on specified network ports or try to keep the connection with given servers.

The application layer is for the instrument driver, TEDS and other user applications, depending on the connected instrument and functions.

### 3.3. Laboratory Test

**Figure 10:**
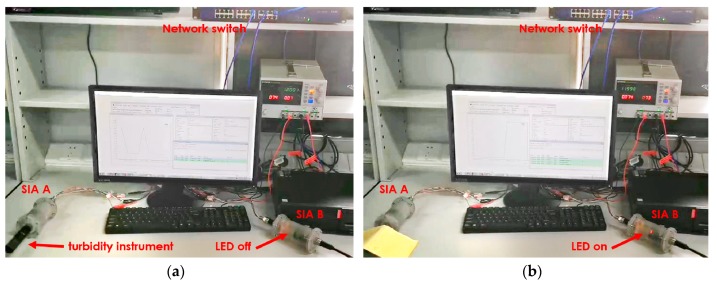
Photos of laboratory test setups: (**a**) The SIAs are just powered up; (**b**) The instrument connected to the SIA A is shaded.

To verify the interoperable capability of the scheme, two SIAs and one PC are used in our laboratory test, as Figure 10 shows. For demonstration purposes, SIAs are encased in 3D-printed resin housings, which will be manufactured by stainless steel or titanium alloy before being deployed in the ocean environment. The SIAs and the PC are interconnected through a network switch with no DHCP server. An MQTT broker starts up on the PC and registers an “_mqtt._tcp” service instance.

To demonstrate the interface standardization method with SIA firmware, a turbidity instrument and an LED are selected, for example, and two drivers are developed for them, respectively. The turbidity instrument has an underwater optical sensor with analog output, which varies between 0 V and 5 V, and 0 V for low turbidity. Once shadowing the detection window to simulate a high turbidity condition, the output signal will be higher. The I/O-controlled LED can be considered as an actuator in the ocean environment. 

The SIA A is connected with the turbidity instrument. After initialization, the driver searches for the MQTT service instance through the DNS-SD and connects to the MQTT broker on the PC. Then it samples the analog output from the instrument and publishes the voltage data to the MQTT broker periodically. The data is also registered to the CoAP server.

The SIA B is used to verify the cooperation among instruments. After initialization, it connects to the MQTT broker in the same way as the SIA A. Then, it searches for instruments through the DNS-SD. For each instrument found, the SIA B queries its PUCK server to search for instruments named “Turbidity Meter”, and subscribes to their data through the MQTT. When the value exceeds the preset threshold, it will turn on the LED on the controller board as cooperative event detection. The status of the LED is registered to the CoAP server module: 0 for off and 1 for on. 

**Figure 11:**
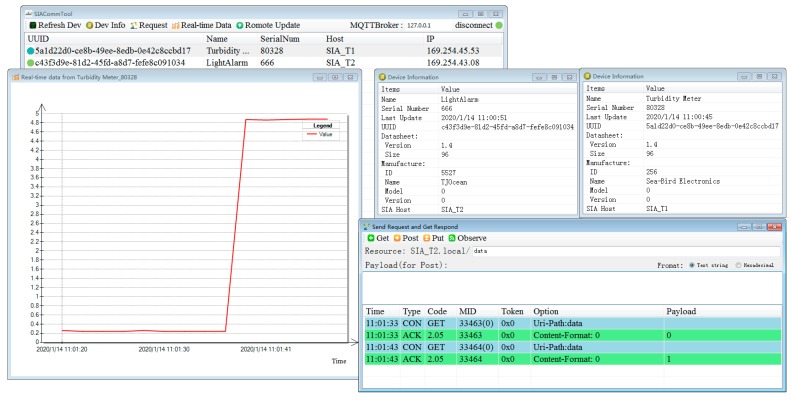
Screenshot of the PC software during the laboratory test.

A software implementing the whole proposed scheme has been developed. Figure 11 is a screenshot of the software in the test. It is developed by the C# language in Visual Studio 2012. After launch, the software follows the common interaction process described in Chapter 2.3 to periodically detect instruments on the network, and the user can also click the “Refresh Dev” button to force detecting. All instruments found will be displayed in the list view. Users can select an instrument in the list view and click the “Dev Info” button to see the detail information, the “Request” button to open request form, in which you can send the CoAP request for resources and see the response, and the “Real-time Data” button to open real-time data form, in which you can see the real-time data published from instruments through the MQTT broker.

The left photo of Figure 10 is when we just powered up SIAs. The PC software finds two instruments and retrieves their information, and the instrument data is received in real-time and displayed. At this moment, the voltage value is close to 0, so that the LED on the SIA B is off. The right photo of Figure 10 shows the status when we shade the optic window of the instrument to simulate the situation that the turbidity increases. We can see from the photo that the LED in SIA B is on. Figure 11 is the screenshot at this moment, we can see the status changes.

The test demonstrates how the SIA firmware standardizes various instrument interfaces and enables the automatic integration of instruments. The host software can recognize the instruments in the same primitive network and interact with them without manual assistance. The collaborative experiment between two instruments proves the capability of machine-to-machine interoperability of the proposed scheme.

## 4. Conclusions

Many ocean observatories are deployed in harsh deep-sea environments where connectivity to the shore is limited. With the increasing number of science instruments in ocean observatories, a flexible, robust, and capable scheme is demanded to integrate ocean instruments. Less manual configuration promises a more robust system and supporting machine-to-machine interaction can maximize scientific research outputs, especially in resource-constrained situations. However, ocean instruments are featured by diverse interfaces, leading to great challenges of achieving automatic instrument integration and machine-to-machine interaction. To deal with these challenges, a systematic instrument interoperable scheme is proposed to standardize instrument interfaces and to enable automatic configuration, service detection, service identification, and standard session even in a rough and temporary local network with limited bandwidth. The proposed scheme integrates several existing standard protocols, i.e., the Zeroconf, PUCK, CoAP, and MQTT protocols, as well as the smart interface module SIA, to achieve instrument plug-and-play and standard communication among heterogeneous ocean instruments. The Zeroconf is used to support automatic network configuration and service detection without external information. Service identification is achieved by employing the PUCK protocol which defines the format of a simple TEDS for instrument recognition. The CoAP and MQTT protocols are selected to implement request/response and publish/subscribe methods, respectively, optimized for resource-constrained scenarios. The scheme specifically considers resource-constrained ocean observatories and machine-to-machine interoperability, which is of great significance to interoperable ocean observatories. The laboratory tests verify the proposed scheme.

The scheme still needs further study on extended metadata to describe software interfaces and instrument features to achieve higher level automatic integration. Future work will be focused on two aspects. One is to test the performance of the scheme in different network conditions, so that we can quantitate the performance of the scheme. The other is to describe the capability, characteristic, demand available, and data format of instruments, i.e., to describe and retrieve the metadata. Finally, a comprehensive software stability test should be carried out and the whole system should be tested in the ocean over a long time period.

## Figures and Tables

**Table 1:** Comparison of PUCK firmware, SEISI and SIA schemes.

Items	PUCK Firmware	SEISI	SIA
Metadata standards	PUCK with others as needed	PUCK, Sensor Model Language (SensorML) and Observations and Measurements(O&M)	PUCK with others as needed
Interaction standards after metadata obtained	Undefined, passed by after metadata obtained	OGC-SWE standards	Constrained Application Protocol (CoAP) and Message Queuing Telemetry Transport (MQTT)
Electrical interface	EIA-232 or Ethernet	Mixed	Mixed (Ethernet in this paper)
Where to achieve standardization	Host-side	Instrument-side or Host-side	Instrument-side
Resource requirement of MCU	Low	Relatively high	Relatively low
Bandwidth requirement	Low	Relatively high	Relatively low
Intelligence	little, almost a storage device	Customizable	Customizable
Integration mode	Passive	Passive	Passive or active, support direct interaction between SIAs

**Table 2:** Comparison of centralized architectures and SOA for ocean observatories.

Items	Centralized Architectures	SOA
Reliability of the whole system	Low, totally depends on the reliability and connectivity of the master node	High, the failure of one node does not crash the whole system
Controllability	Highly Centralized	Distributed
Scalability	Low	High
Performance requirement of controller	Extremely high for the master node, low for slave nodes.	Higher than that of the slave node in the centralized one.
Connectivity requirement	Always connected with the master node.	Connectivity between any two nodes.
